# Validation of the Dyspnoea-12 and Multidimensional Dyspnea profile among older Swedish men in the population

**DOI:** 10.1186/s12877-022-03166-5

**Published:** 2022-06-02

**Authors:** Max Olsson, Magnus Ekström

**Affiliations:** Faculty of Medicine, Dept of Clinical Sciences Lund, Respiratory Medicine, Allergology and Palliative Medicine, Lund University, 221 85 Lund, Sweden

**Keywords:** Dyspnoea-12, Multidimensional Dyspnea Profile, Breathlessness, Older men, Older adults, Population, Psychometrical properties, Respiratory disease, Cardiovascular disease

## Abstract

**Background:**

The Dyspnoea-12 (D12) and Multidimensional dyspnea profile (MDP) are commonly used instruments for assessing multiple dimensions of breathlessness but have not been validated in older people in the population. The aim of this study was to validate the D12 and MDP in 73-years old men in terms of the instruments’ underlying factor structures, internal consistency, and validity.

**Methods:**

A postal survey was sent out to a population sample of 73-years old men (*n* = 1,193) in southern Sweden. The two-factor structures were evaluated with confirmatory factor analysis, internal consistency with Cronbach's alpha, and validity using Pearson´s correlations with validated scales of breathlessness, anxiety, depression, fatigue, physical/mental quality of life, body mass index (BMI), and cardiorespiratory disease.

**Results:**

A total 684 men were included. Respiratory and cardiovascular disease were reported by 17% and 38%, respectively. For D12 and MDP, the proposed two-factor structure was not fully confirmed in this population. Internal consistency was excellent for all D12 and MDP domain scores (Cronbach's alpha scores > 0.92), and the instruments’ domains showed concurrent validity with other breathlessness scales, and discriminant validity with anxiety, depression, physical/mental quality of life, BMI, and cardiorespiratory disease.

**Conclusions:**

In a population sample of 73-years old men, the D12 and MDP had good psychometrical properties in terms of reliability and validity, which supports that the instruments are valid for use in population studies of older men.

**Supplementary Information:**

The online version contains supplementary material available at 10.1186/s12877-022-03166-5.

## Background

Breathlessness is prevalent among older people [1, 2], with 15% of the population 50 + experiencing breathlessness that limits their daily life [3]. Breathlessness is strongly associated with a multitude of factors including the presence and severity of cardiorespiratory disease [1], anxiety, depression, and obesity [4]. Often, several contributing factors overlap among patients suffering from breathlessness [5]. In many cases the underlying condition causing a patient’s breathlessness is unknown [4]. Breathlessness is a multidimensional symptom and includes physical, emotional, affective and overall unpleasantness dimensions that comprise the perceived symptom [5]. It is important to evaluate several dimensions to better describe and understand the person’s perceptions [6].

The instruments Dyspnoea-12 (D12) [7] and the Multidimensional Dyspnea Profile (MDP) [8] are commonly used to measure multiple dimensions of breathlessness in experimental and clinical observational studies [9, 10]. The instruments are not interchangeable [11]. The D12 and MDP has been validated for use across multiple languages and conditions and shows overall a high internal consistency and external validity [9, 12, 13].

However, knowledge is limited on the psychometric properties of the D12 and MDP in larger populational studies of older adults [9, 14]. To date, validation studies of D12 and MDP were conducted mainly in disease selected cohorts [9]. One study [14] used a small sample to validate the MDP among older adults, however, the sample was not drawn from the general older population. The distribution of breathlessness dimensions in the previous validation studies are unbalanced, and mainly include participants with severe breathlessness and chronical diseases, such as chronic obstructive pulmonary disease (COPD) and interstitial lung disease (ILD) [9]. The psychometrical properties of D12 and MDP are therefore unknown and can be less valid among individuals with mild breathlessness, and in people in a general population which may have fewer and less severe underlying conditions [14]. Compared to studies of severe underlying conditions, the instruments can be less valid when evaluating the burden of public health issues that are associated with breathlessness [4, 9], such as obesity, anxiety, and depression.

The aim of this study was to validate the D12 and MDP for use in populational studies of older adults in terms of underlying factor structure, internal consistency, and validity.

## Material and methods

### Study design and population

The VAScular and Chronic Obstructive Lung disease (VASCOL) study started in 2011 as a population level longitudinal cohort study of 65-years-old males (*n* = 1,302). The men were recruited from a screening campaign of abdominal aortic aneurysm (AAA), which was offered to all men at the age of 65 in 2011–2012, living in the county of Blekinge, Sweden. At the same time the men were invited to the screening campaign, they were also invited to participate in the VASCOL study. In 2019, all participants who were alive and had a known address (*n* = 1,193) were invited to a postal follow-up survey, focused on patient reported outcomes including breathlessness. The men were approximately 73 years old at this time and the data from this follow-up study was used in the present study. The VASCOL data collection and a lost to follow-up analysis of the men not attending the present study is described in the protocol article for the VASCOL study [15]. At the baseline, the men participating in the follow-up had a lower proportion of current smokers and lung obstruction, a lower average pack-years of smoking, and had overall higher education compared to the men not participating in the follow-up [15]. Inclusion criteria for the present study were participants that reported data on the D12 and MDP scores. The database was previously used to study the prevalence of multiple dimensions of breathlessness [16], and of their relation to health related quality of life (HrQoL) [17].

### Assessments

The following assessments were self-reported by the participants: height, weight, body mass index (BMI), smoking status, pack-years of smoking, and physician diagnosed lung disease (COPD, asthma*,* tuberculosis, sleep apnoea, or other lung disease), cardiovascular disease (myocardial infarction, angina, atrial fibrillation, heart failure, valvular heart, bypass, aortic aneurysm, carotid artery stenosis*,* or stroke) and diabetes mellitus. The participants were also asked to state how frequent they exercised (at least 30 min) with the possible answers: *every day*, *3–6 times a week*, *1–3 times a week*, and *less than once a week* [15].

The Swedish version of D12 and MDP used have been validated to be used in postal surveys in patients with cardiorespiratory diseases [12, 13], and the instruments has also been validated among older adults [14], however in non-population cohort. The D12 comprises 12 descriptors of breathlessness which is scored by the participant as “*None*”, “*Mild*”, “*Moderate*”, or “*Severe*”. A total score can be summarised (range 0—36), as well as a physical (maximum 21) and affective (range 0—15) domain score [7]. The MDP consist of 11 descriptors of breathlessness rated using numerical rating scales (NRS) ranging from 0 to 10. A total score can be summarised as well as an immediate perception subdomain (6 items), and an emotional response subdomain (5 items). If the MDP should be used to present an overall experience of breathlessness, it is recommended to use the MDP A1 unpleasantness item (NRS 0–10) [8].

The validity of the D12 and MDP were evaluated against the following self-reported measurements: breathlessness using the modified Medical Research Council (mMRC) scale [18], and the item for current breathlessness intensity (NRS 0–10) of Edmonton Symptom Assessment System Revised (ESAS-r) [19], BMI, HrQoL using the Short form 12 item (version 2) physical and mental health composite score (SF-12 PCS; SF-12 MCS) [20], anxiety and depression using the Hospital Anxiety and Depression Scale (HADS) [21], and fatigue using the FACIT-Fatigue [22]. The focal period for all instruments were “the last two weeks”.

### Statistical analysis

Descriptive statistics were used for presenting participants characteristics. The original proposed two-factor structures [7, 8] of the D12 (physical and affective factor structure) and MDP (immediate perception and emotional response factor structure) were evaluated using confirmatory factor analysis (CFA), and then plotted. The goodness of fit of the CFA was evaluated by the root mean square error of approximation (RMSEA) [23] and Bentler´s comparative fit index (CFI) [24]. To compare the factor structure of the D12 and MDP among the total population sample with participants with cardiorespiratory disease, CFA were also performed among participants with cardiovascular disease and/or lung disease.

Internal consistency of the D12 and MDP total and subdomain scores were evaluated using Cronbach's alpha using the following thresholds to describe the result: < 0.5 = Unacceptable; > 0.5 = Poor; > 0.6 = Questionable; > 0.7 = Acceptable; > 0.8 = Good; > 0.9 = Excellent [25]. To evaluate the robustness of the D12 and MDP total if items would be dropped, items were removed one-by-one and the effects on D12 and MDP totals’ Cronbach´s alpha estimates were presented.

Pearson´s correlation coefficients were used to evaluate the validity of the D12 and MDP scores were evaluated with: mMRC, ESAS-r breathlessness scale, SF-12 (PCS and MCS), HADS (total, anxiety, and depression score), the FACIT-Fatigue total score, BMI, presence of respiratory disease, cardiovascular disease. The Pearson´s correlation coefficients were then plotted, and the following thresholds were used to describe the magnitude of the correlation (negative): 0.70 to 0.9 (-0.70 to -0.9) was considered as high correlation and 0.5 to 0.69 (-0.5 to -0.69) was considered as moderate correlation. Low correlation was considered as 0.3 to 0.49 (-0.3 to -0.49), and 0 to 0.30 (0 to -0.30) was considered as negligible correlation [26]. When measuring validity of an instrument, the COnsensus-based Standards for the selection of health Measurement Instruments (COSMIN) [27] recommends studies to state the expected relationships with other outcomes measures. All D12 and MDP domains were expected to have moderate to high positive correlation between each other and with ESAS-r breathlessness scale, respiratory disease, and cardiovascular disease. D12 physical and MDP immediate perception were expected to have a high positive correlation with mMRC and BMI, a moderate positive correlation with HADS (total, anxiety, and depression score), a high negative correlation with SF-12 PCS, and moderate negative correlation with SF-12 MCS and Facit fatigue. D12 affective and MDP emotional response were expected to have a high positive correlation with HADS (total, anxiety, and depression score), a moderate positive correlation with mMRC and BMI, a high negative correlation to SF-12 MCS, and a moderate negative correlation with SF-12 PCS and Facit fatigue. Statistical analysis was conducted with R 4.0.2 (R Foundation for Statistical Computing, Austria).

## Results

Out of the 1193 men invited, 907 (76%) men returned the survey, and 684 (57%) men provided data on all D12 and MDP items and were included in the present analysis. Participant characteristics are shown in Table 1; mean BMI 27.2 (standard deviation [SD] 3.9) and 445 (65%) were current or former smokers, and pack-years of smoking mean was 9 (SD 14.1). The prevalence of breathlessness (mMRC ≥ 2) was 18%. At least one respiratory disease was reported by 17%, and at least one cardiovascular disease was reported by 38%, Table 1. The D12 total mean was 1.7 (SD 4.2) and the MDP A1 mean 0.7 (SD 1.4) (Table 2). The frequency distribution of the D12 and MDP items are shown in Supplementary Table S1 and Supplementary Table S2.

**Table 1:** Characteristics of men aged 73 years old in the population (*n* = 684)

Variable (non-missing observations)	Mean (SD) or Frequency (%)
BMI, kg/m2 (*n* = 677)	27.2 (3.9)
Smoking status (*n* = 676)	
Daily	34 (5%)
Sometimes	7 (1%)
Former smoker	404 (60%)
Never smoker	231 (34%)
Exercise at least 30 min (*n* = 677)	
Every day	172 (25%)
3–6 times a week	205 (30%)
1–3 times a week	192 (28%)
Less than once a week	108 (16%)
Pack-years of smoking (*n* = 634)	9 (14.1)
Lung disease (*n* = 654)^*^	111 (17%)
COPD	26 (4%)
Asthma	35 (5%)
Cardiovascular disease (*n* = 654)^**^	246 (38%)
Myocardial infarction	66 (10%)
Angina	49 (7%)
Atrial fibrillation	105 (16%)
Heart failure	27 (4%)
Stroke	51 (8%)
Diabetes mellitus (*n* = 654)	96 (15%)

Data is presented as either mean (SD) or frequencies (%)

*BMI* Body mass index, *COPD* chronic obstructive pulmonary disease

^*^COPD, asthma, tuberculosis, sleep apnoea, or other lung disease

^**^Myocardial infarction, angina, atrial fibrillation, heart failure, valvular heart, bypass, aortic aneurysm, carotid artery stenosis, or stroke

**Table 2:** Participants reported outcomes of men aged 73 years old (*n* = 684)

Variable (non-missing observations)	Mean (SD) or Frequency (%)
**D12 (*n* = 684)**	
Total	1.7 (4.2)
Physical domain score	1.1 (2.5)
Affective domain score	0.6 (1.8)
D12, respiratory disease (*n* = 111) *	
Total	3.6 (6.4)
Physical domain score	2.4 (3.8)
Affective domain score	1.3 (2.9)
D12, cardiovascular disease (*n* = 246) **	
Total	2.4 (5.2)
Physical domain score	1.6 (3.1)
Affective domain score	0.9 (2.3)
**MDP (*n* = 684)**	
A1 unpleasantness score	0.7 (1.4)
Immediate Perception	2.7 (6.5)
Emotional response	1.8 (5.1)
MDP, respiratory disease (*n* = 111) *	
A1 unpleasantness score	1.3 (2.0)
Immediate Perception	5.4 (9.9)
Emotional response	2.8 (5.9)
MDP, cardiovascular disease (*n* = 246) **	
A1 unpleasantness score	1.0 (1.7)
Immediate Perception	3.8 (8.3)
Emotional response	2.3 (5.8)
**mMRC class (*n* = 668)**	
0	453 (68%)
1	98 (15%)
≥ 2	117 (18%)
ESAS-r breathlessness scale (*n* = 672)	1.8 (2.4)
SF12 PCS (*n* = 672)	46.7 (9.0)
SF12 MCS (*n* = 672)	54.4 (8.9)
HADS total (*n* = 642)	6.4 (5.9)
HADS depression (*n* = 664)	3 (3.1)
HADS anxiety (*n* = 657)	3.4 (3.5)
Facit fatigue (*n* = 677)	42 (8.9)

*D12* Dyspnoea-12, *MDP* multidimensional dyspnoea profile, *mMRC* Modified Medical Research Council dyspnea scale, *ESAS-r* Edmonton Symptom Assessment System. Revised, *HADS* Hospital Anxiety and Depression Scale, *SF12 PCS* Short form 12 item (version 2) physical health composite score, *SF12 MCS* Short form 12 item (version 2) mental health composite score

*COPD, asthma, tuberculosis, sleep apnoea, or other lung disease

**Myocardial infarction, angina, atrial fibrillation, heart failure, valvular heart, bypass, aortic aneurysm, carotid artery stenosis, or stroke

**Fig. 1:**
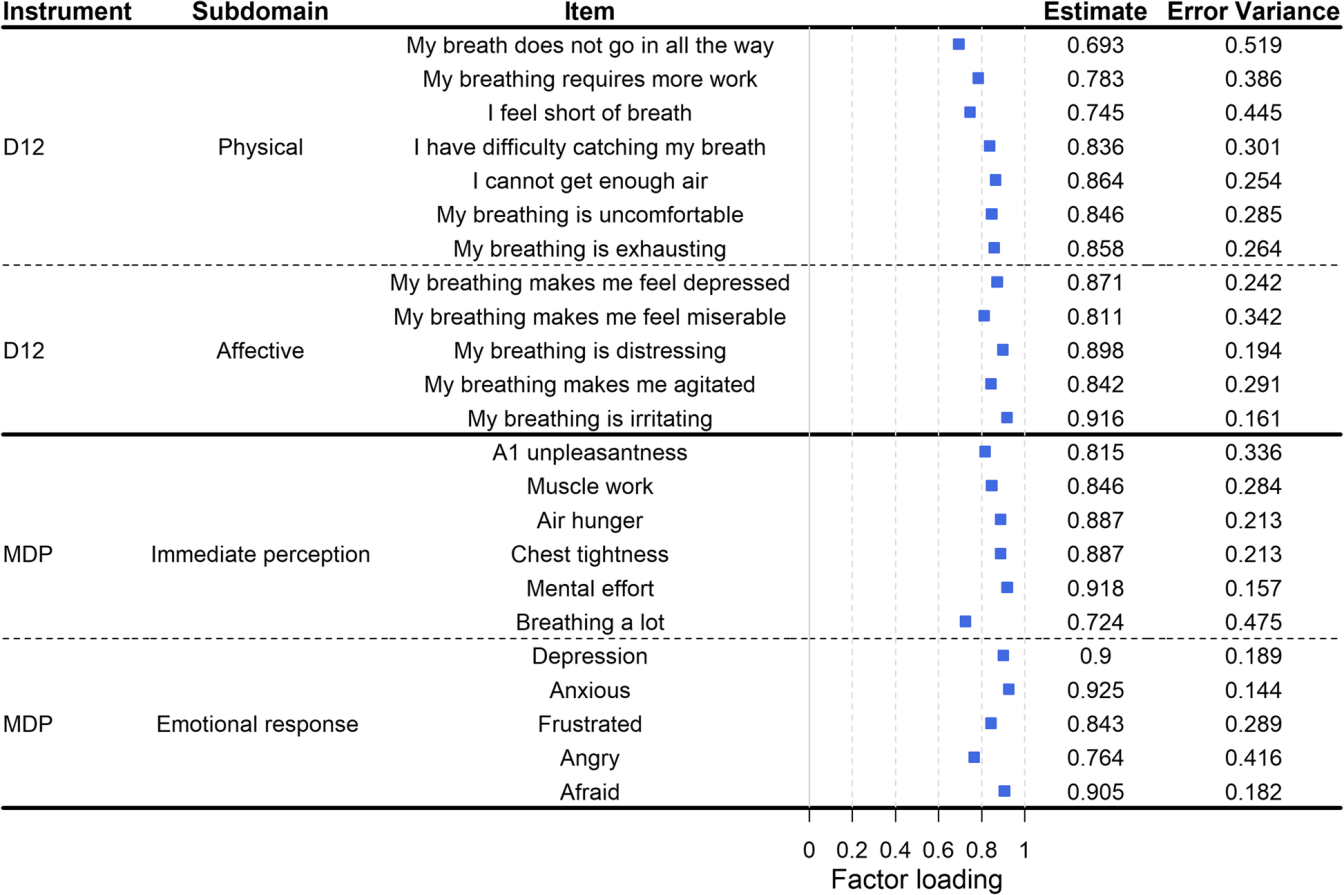
Confirmatory factor analysis for Dyspnoea-12 and Multidimensional Dyspnea Profile. Confirmatory factor analysis for the two-factor structure of Dyspnoea-12 and Multidimensional Dyspnea Profile, respectively. *Estimate* corresponds to the factor loading of each item and a higher factor loading means the item relates more to it’s given subdomain compared to a lower factor loading. *Error variance* corresponds to how much of the variance in each item is not covary with the given domain. All values are standardized. *D12* Dyspnoea-12, *MDP* Multidimensional Dyspnea Profile

Factor structure analyses using CFA are shown in Fig. 1. The two-factor structures were not fully confirmed as the CFA model fit was sub-optimal for D12 (RMSEA = 0.137, CFI = 0.914) and MDP (RMSEA = 0.134, CFI = 0.928). The D12 “*My breath does not go in all the way*” and MDP “*Breathing a lot*” showed a lower factor loading than the other items (Fig. 1). The factor loadings and the CFA model fits of the D12 and MDP were similar among participants with cardiorespiratory diseases compared to the total participant sample (Supplementary Table S3; Supplementary Table S4).

Internal consistency was excellent for the D12 and MDP total score and subdomain scores, all having a Cronbach´s alpha score of > 0.92, as shown in Table 3. When single items were removed one-by-one, the D12 total and MDP total Cronbach´s alpha estimate only changed minimally (D12 total ≤ 0.007; MDP total ≤ 0.008), meaning that the internal consistency of the D12 total and MDP A1 scores were robust when removing individual item scores.

**Table 3:** Internal consistency of Dyspnoea-12 and Multidimensional Dyspnea Profile scores

Variable	Cronbach’s alpha estimate
D12 total	0.956
D12 physical domain	0.924
D12 affective domain	0.936
MDP total	0.943
MDP immediate perception subdomain	0.932
MDP emotional response subdomain	0.939

Cronbach’s alpha estimate for 73-years old men in the population (*n* = 684). *D12* Dyspnoea-12, *MDP* Multidimensional dyspnea profile scores

**Fig. 2:**
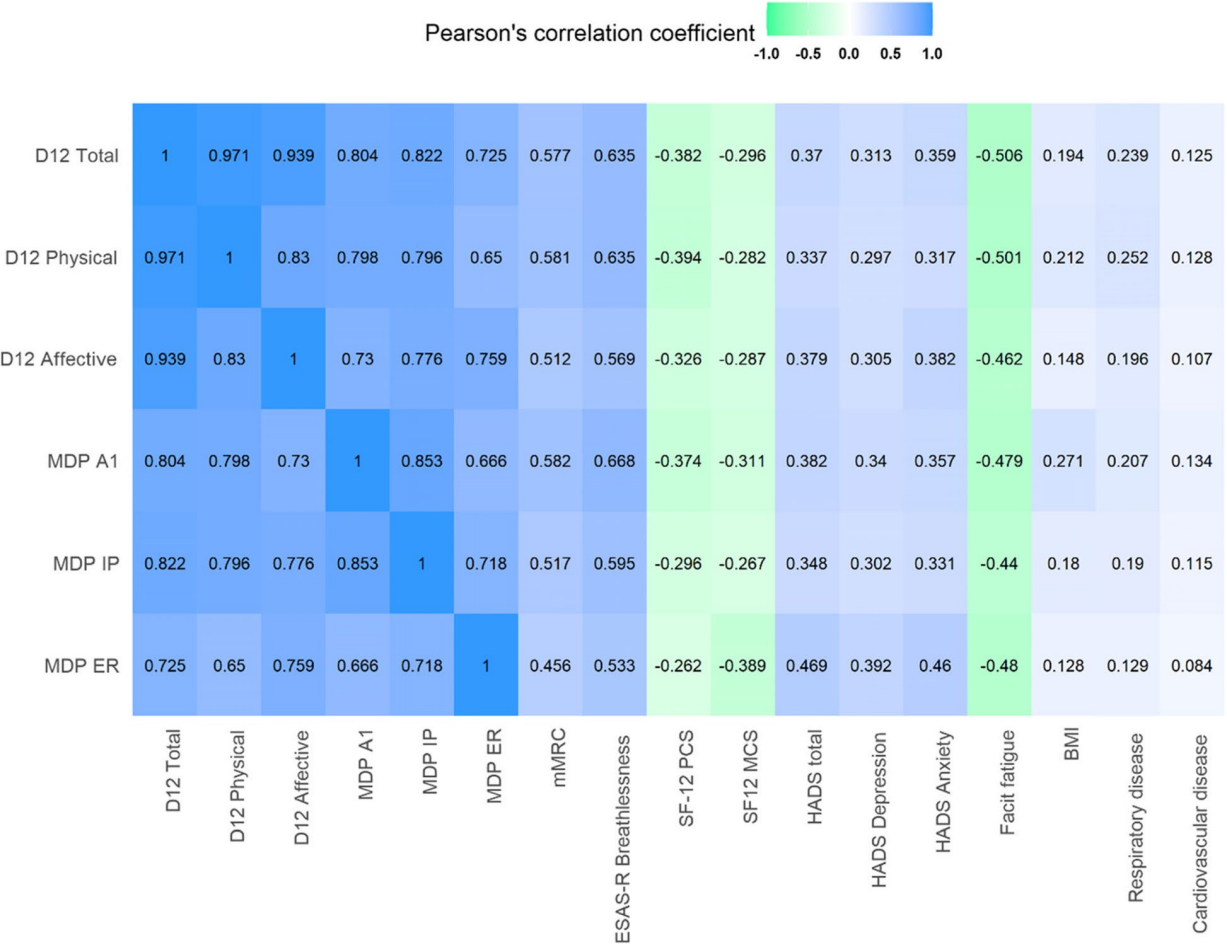
Validity for Dyspnoea-12 and Multidimensional Dyspnea Profile among 608 participants. Validity between Dyspnoea-12 and Multidimensional dyspnea profile measured with Pearson’s correlation coefficient between scales. The 95% confidence intervals are presented in parenthesis below the coefficients. The strength of correlation between the scales is represented with colour intensity, and higher colour intensity means stronger positive or negative correlation. *Abbreviations: BMI* Body mass index, *D12* Dyspnoea-12, *MDP* multidimensional dyspnoea profile, *IP* Immediate Perception, *ER* Emotional Response, *mMRC* Modified Medical Research Council dyspnea scale, *ESAS-r* Edmonton Symptom Assessment System. Revised, *HADS* Hospital Anxiety and Depression Scale, *SF-12 PCS* Short form 12 item (version 2) physical health composite score, *SF-12 MCS* Short form 12 item (version 2) mental health composite score

Concurrent validity estimates are shown in Fig. 2. The D12 total and MDP A1 scores was highly correlated between each other. The D12 physical score was highly correlated with the MDP immediate perception, and the D12 affective was highly correlated with MDP emotional response. The D12 physical and affective subdomains, as well as the MDP A1 and immediate perception were similar moderately correlated with the other breathlessness scales mMRC and ESAS-r. The MDP emotional response was moderately correlated with ESAS-r and weakly correlated with mMRC, (Fig. 2). The D12 affective, MDP A1, and MDP emotional response were more strongly correlated with the HADS total, HADS depression, HADS anxiety, and SF-12 MCS, compared to the D12 physical and MDP immediate perception. At the same time, the D12 physical and MDP immediate perception were overall more strongly correlated with the mMRC, SF-12 PCS, and BMI compared to the D12 affective, MDP A1, and MDP emotional response (Fig. 2). The D12 and MDP were weakly to moderately correlated to FACIT fatigue. All D12 and MDP scores were more strongly correlated to the presence of respiratory disease compared to the presence cardiovascular disease, and the D12 physical and MDP immediate perception were more strongly correlated to the presence of respiratory disease compared to D12 affective and MDP emotional response (Fig. 2).

## Discussion

### Main findings

The main finding is that the D12 and MPD are valid for measuring multiple dimensions of breathlessness in population surveys of 73-years old men. The psychometric properties were similar to those reported in a systematic review of D12 and MDP [9]. The D12 and MDP showed validity between each other and with other breathlessness scales, anxiety/depression, fatigue, BMI, and physical and mental HrQoL. Both instruments had excellent internal consistency and validity in this populational study which strengthens the usefulness of the instruments in large epidemiological studies of older men. However, the original proposed two-factor structures were not fully confirmed.

### What this study adds

This study extends previous validation data that D12 and MDP are valid for use in future epidemiological studies or surveys of breathlessness among 73-years old men. The instruments had concurrent validity by showing expected associations with other important patient-reported outcomes. The discriminant validity of the instruments with BMI and physical and mental HrQoL is a novel finding which can be valuable for public health studies. We presented the largest populational sample reporting the D12 and MDP so far [11] and included participants with and without multiple different conditions.

The high Cronbach´s alpha estimates support that the domains of both instruments are reliable. The Cronbach´s alpha estimates were very similar to the pooled estimates of 18 D12 studies in a large international systematic review, and our study had a higher Cronbach´s alpha estimate than the pooled estimate of nine MDP studies [9].

In contrast to the original validation studies, the proposed two-factor structures of D12 and MDP were not fully confirmed in this study, as the fit of the CFA models were not optimal. The items “*My breath does not go in all the way*” (D12 physical) and “*Breathing a lot*” (MDP immediate perception) showed a lesser factor loading than the rest of the items, and means that they do not fully belong to the original proposed factor structure and domains (D12 physical; MDP immediate perception) [7, 8]. Older people can experience these items (descriptors) as being more emotional/affective loaded in comparison to the participants in the original studies confirming the two-factor structures [7, 8, 28]. A previous study evaluating the MDP among older adults also reported variations in the factor loading estimate of the items [14]. Further, the affective/emotional domains also shows correlation with the physical/immediate perception in this and a previous study [29], and this can explain some items’ lower factor loading, as some items can “leak” into the other domain. The original validation study proposing the factor structure of the D12 and MDP included patients with cardiorespiratory diseases [7, 28] in comparison to our population sample that included participants with, and without various conditions. The factor loadings were similar among cardiorespiratory participants and the total population sample in our study. Our population has larger diversity of underlying conditions present compared to the original validation studies, which can explain lower factor loadings, as the experiences can be more different from each other, compared to participants with the same or similar conditions. Previous studies suggests that different physiological conditions can lead to different experiences of breathlessness [30], and with our result in mind, the original factor structures can be less valid for participants with various conditions. Future studies should evaluate the original proposed factor structures among participants with common conditions associated with breathlessness such as obesity, and anxiety/depression. Future studies can also use exploratory factor analysis (EFA) or Exploratory Structural Equation Modelling (SEM) to explore alternative factors structures of the D12 and MDP among individuals with various conditions. When comparing the fit of the CFA to studies using the same Swedish version of the instruments, the MDP showed a similar sub-optimal fit [13], but the D12 showed worse fit in our study [12]. The previous studies used a population more similar to the original studies populations [7, 8] with cardiorespiratory participants and overall more severe breathlessness [12, 13], which supports the worse CFA fit for the D12 in our study.

The factor structure of the D12 and MDP has only been confirmed in five and six studies, respectively, in comparison to 27 studies evaluating the internal consistency of the instruments [9]. The relative paucity of data and our failure to fully confirm the factor structure warrants further investigations of the underlying factor structure of each instrument and of the independence (of each other) and utility (compared with the summary score) of the proposed factors.

A novel finding of our study is that we found discriminant validity for the D12 and MDP subdomains for BMI, and mental and physical HrQoL. We also found that the correlation of D12 physical and MDP immediate perception were stronger to respiratory disease compared to D12 affective and MDP emotional response, and this can reflect the physical limitations a respiratory disease induces for the individual. This new knowledge of the discriminant validity of the D12 and MDP can be helpful in epidemiological studies, especially as physical and mental HrQoL, BMI and limitations of conditions are important outcomes in public health research. The strength of the correlations between the D12 and MDP subdomains reflects what they intend to measure: one physical/perception and one affective/emotional domain in each instrument. This is especially clear as increased D12 physical and MDP perception subdomains being negatively correlated to better physical HrQoL, and increased D12 affective and MDP emotional subdomains being negatively correlated to mental HrQoL. Even though the correlations are fairly strong between the domains of the D12 and MDP, they were not perfectly correlated, and this supports that the instruments are measuring different aspects of breathlessness and are not interchangeable, as suggested before [11]. The instruments were overall moderate correlated with the other breathlessness scales mMRC and ESAS-R breathlessness scale, which supports the concurrent validity of the D12 and MDP. The correlation in-between the D12 and MDP scores were similar to a previous study of COPD patients [11]. The D12 and MDP subdomains correlations with the mMRC, HADS anxiety and depression in our study are very similar to the pooled estimates in a recent systematic review of the D12 and MDP [9]. The D12 physical subdomain were more strongly correlated with Facit fatigue compared to the affective subdomain, but this is different from another study which did not show any differences between the subdomains’ correlation to Facit fatigue [12]. The MDP immediate perception was more strongly correlated to Facit fatigue compared to emotional response, which is different from a previous study [13]. However, as the differences of the strength of correlations between the subdomains and Facit fatigue were not big in our study nor in the previous studies [12, 13], it is hard to make any conclusions as now. Overall, the evaluation of the validity reinforces the D12 and MDP as useful instruments when assessing multiple dimensions of breathlessness in epidemiological studies of older men´s health.

### Strengths and limitations

The study has several strengths. It used a large sample size, and the study includes participants with and without a diverse set of clinical conditions which yields better generalizability. Also, the study used a vast number of validated instruments and patient outcomes for the evaluation of the validity, which strengthen the validity of the study. The study´s result is similar to a systematic review of both instruments psychometrical properties [9], which strengthen the reliability of the study. The present study also has some limitations. The majority of the participants had none or mild breathlessness (mMRC ≤ 1) and scored low on the D12 and MDP items. The instruments can therefore be less valid for participants with severe breathlessness. Still, 117 of the participants were moderate to severe breathless (mMRC ≥ 2) which is a larger sub sample than the majority of the previous D12 and MDP validation studies’ total sample sizes [9]. The prevalence of breathlessness was similar to another study of older adults [2], and our result can therefore be relevant for studies using the D12 and MDP to measure breathlessness among similar aged men in the population. Since the population only included 73 years old men we cannot generalise for women and individuals in younger age groups and there is a need of further validation studies including comparison between men and women, and between age groups. Lastly, the cross-sectional study design does not allow evaluation of test–retest reliability of the instruments, which is an important measurement for repeatability.

## Conclusions

The D12 and MDP have good psychometrical properties in terms of reliability and validity and should be seen as usable in population studies to assess multiple dimensions of breathlessness among 73 years old men. A novel finding is that the instruments have discriminant validity with BMI, mental and physical HrQoL, and respiratory disease, which are common outcomes in public health studies. However, the original factor structures were not fully confirmed in this study. Future studies should further evaluate the factor structures of the D12 and MDP and the instruments psychometrical properties in common conditions associated with breathlessness such as obesity, anxiety, and depression.

## Supplementary Information

**Additional file 1:** **Supplementary Table S1. **Frequency distribution ofDyspnoea-12 item scores among 684 participants. **Supplementary Table S2.**Frequency distribution of Multidimensional dyspnea profile item scores among684 participants. **Supplementary Table S3.**Confirmatory factor analysis forDyspnoea-12 among 312 participants with cardiorespiratory diseases. **SupplementaryTable S4. **Confirmatory factor analysis for Multidimensional Dyspnea Profileamong 312 participants with cardiorespiratory diseases.

## Data Availability

The dataset used in this study is available from the corresponding author upon reasonable request. New study objectives must be approved by the Sweden’s national ethical review board.

## Abbrevations

BMI: Body mass index
CFA: Confirmatory factor analysis
CFI: Comparative fit index
CI: Confidence intervals
COPD: Chronic obstructive pulmonary disease
D12: Dyspnoea-12
EFA: Exploratory factor analysis
ESAS-r: Edmonton Symptom Assessment System Revised
HADS: Hospital Anxiety and Depression Scale
HrQoL: Health related quality of life
ILD: Interstitial lung disease
MDP: Multidimensional dyspnea profile
mMRC: modified Medical Research Council breathlessness scale
NRS: Numerical rating scales
RMSEA: Root mean square error of approximation
SEM: Structural Equation Modelling
SF-12 MCS: Short form 12 item mental health composite score
SF-12 PCS: Short form 12 item physical health composite score
VASCOL: VAScular and Chronic Obstructive Lung disease
