# Supplementary material for: Validation of the Dyspnoea-12 and Multidimensional Dyspnea profile among older Swedish men in the population
Source: BMC Geriatr. 2022 Jun 2;22:477. doi: 10.1186/s12877-022-03166-5 (PMC9164708; doi:10.1186/s12877-022-03166-5)
Supplement: Supplementary file 1 — Additional file 1: Supplementary Table S1. Frequency distribution ofDyspnoea-12 item scores among 684 participants. Supplementary Table S2.Frequency distribution of Multidimensional dyspnea profile item scores among684 participants. Supplementary Table S3.Confirmatory factor analysis forDyspnoea-12 among 312 participants with cardiorespiratory diseases. SupplementaryTable S4. Confirmatory factor analysis for Multidimensional Dyspnea Profileamong 312 participants with cardiorespiratory diseases. [file 12877_2022_3166_MOESM1_ESM.docx]

**Supplementary Material -** **Validation of the Dyspnoea-12 and Multidimensional Dyspnea Profile among older Swedish men in the population.**

**Supplementary Table S1. Frequency distribution of Dyspnoea-12 item scores among 684 participants.**

|  | **Category** | | | |
| --- | --- | --- | --- | --- |
| **Item** | **None (0)** | **Mild (1)** | **Moderate (2)** | **Severe (3)** |
| My breath does not go in all the way | 623 | 46 | 12 | 3 |
| My breathing requires more work | 574 | 97 | 12 | 1 |
| I feel short of breath | 515 | 142 | 21 | 6 |
| I have difficulty catching my breath | 599 | 67 | 16 | 2 |
| I cannot get enough air | 597 | 74 | 12 | 1 |
| My breathing is uncomfortable | 629 | 40 | 12 | 3 |
| My breathing is exhausting | 626 | 44 | 13 | 1 |
| My breathing makes me feel depressed | 627 | 44 | 11 | 2 |
| My breathing makes me feel miserable | 648 | 26 | 8 | 2 |
| My breathing is distressing | 608 | 56 | 17 | 3 |
| My breathing makes me agitated | 635 | 34 | 12 | 3 |
| My breathing is irritating | 599 | 61 | 21 | 3 |

Numbers in the table´s cells correspond to number of participants.

**Supplementary Table S2.** **Frequency distribution of Multidimensional dyspnea profile item scores among 684 participants.**

|  | **Numerical rating scale (0 – 10)** | | | | | | | | | | |
| --- | --- | --- | --- | --- | --- | --- | --- | --- | --- | --- | --- |
| **Item** | **0** | **1** | **2** | **3** | **4** | **5** | **6** | **7** | **8** | **9** | **10** |
| A1 unpleasantness | 460 | 104 | 54 | 37 | 6 | 11 | 3 | 6 | 3 | 0 | 0 |
| Muscle work | 582 | 43 | 19 | 19 | 6 | 6 | 4 | 2 | 2 | 0 | 1 |
| Air hunger | 593 | 44 | 17 | 10 | 3 | 6 | 4 | 4 | 1 | 2 | 0 |
| Chest tightness | 582 | 47 | 21 | 11 | 7 | 4 | 5 | 1 | 3 | 2 | 1 |
| Mental effort | 607 | 34 | 18 | 9 | 3 | 5 | 4 | 0 | 1 | 2 | 1 |
| Breathing a lot | 535 | 67 | 28 | 14 | 12 | 11 | 5 | 5 | 1 | 3 | 3 |
| Depression | 612 | 30 | 16 | 8 | 7 | 6 | 1 | 0 | 2 | 1 | 1 |
| Anxious | 576 | 48 | 29 | 11 | 7 | 6 | 2 | 2 | 1 | 1 | 1 |
| Frustrated | 571 | 40 | 27 | 20 | 6 | 13 | 3 | 1 | 1 | 1 | 1 |
| Angry | 588 | 44 | 19 | 16 | 4 | 6 | 4 | 0 | 1 | 1 | 1 |
| Afraid | 584 | 51 | 17 | 8 | 7 | 9 | 2 | 1 | 2 | 2 | 1 |

Numbers in the table´s cells correspond to number of participants. Higher item score indicates increased severity.

**Supplementary Table S3.** **Confirmatory factor analysis for Dyspnoea-12 among 312 participants with cardiorespiratory diseases.**

| **Subdomain** | **Item** | **Factor loading** | **Error Variance** |
| --- | --- | --- | --- |
| Physical | My breath does not go in all the way | 0.697 | 0.515 |
| Physical | My breathing requires more work | 0.772 | 0.404 |
| Physical | I feel short of breath | 0.759 | 0.424 |
| Physical | I have difficulty catching my breath | 0.853 | 0.272 |
| Physical | I cannot get enough air | 0.839 | 0.296 |
| Physical | My breathing is uncomfortable | 0.829 | 0.313 |
| Physical | My breathing is exhausting | 0.857 | 0.265 |
| Affective | My breathing makes me feel depressed | 0.904 | 0.182 |
| Affective | My breathing makes me feel miserable | 0.867 | 0.249 |
| Affective | My breathing is distressing | 0.889 | 0.201 |
| Affective | My breathing makes me agitated | 0.840 | 0.294 |
| Affective | My breathing is irritating | 0.904 | 0.183 |

The root mean squared error (RMSE) of the confirmatory factor analysis (CFA) was 0.138 and the confirmatory fit index (CFI) was 0.914. Cardiorespiratory diseases were self-reported as myocardial infarction, angina, atrial fibrillation, heart failure, valvular heart, bypass, aortic aneurysm, carotid artery stenosis, stroke, chronic obstructive pulmonary disease [COPD], asthma, tuberculosis, sleep apnoea, or other lung disease.

**Supplementary Table S4. Confirmatory factor analysis for Multidimensional Dyspnea Profile among 312 participants with cardiorespiratory diseases.**

| **Subdomain** | **Item** | **Factor loading** | **Error Variance** |
| --- | --- | --- | --- |
| Immediate perception | A1 unpleasantness | 0.841 | 0.293 |
| Immediate perception | Muscle work | 0.852 | 0.275 |
| Immediate perception | Air hunger | 0.877 | 0.232 |
| Immediate perception | Chest tightness | 0.891 | 0.205 |
| Immediate perception | Mental effort | 0.917 | 0.160 |
| Immediate perception | Breathing a lot | 0.724 | 0.477 |
| Emotional response | Depression | 0.857 | 0.266 |
| Emotional response | Anxious | 0.888 | 0.212 |
| Emotional response | Frustrated | 0.829 | 0.313 |
| Emotional response | Angry | 0.751 | 0.435 |
| Emotional response | Afraid | 0.885 | 0.217 |

The root mean squared error (RMSE) of the confirmatory factor analysis (CFA) 0.158 and the confirmatory fit index (CFI) was 0.900. Cardiorespiratory diseases were self-reported as myocardial infarction, angina, atrial fibrillation, heart failure, valvular heart, bypass, aortic aneurysm, carotid artery stenosis, stroke, chronic obstructive pulmonary disease [COPD], asthma, tuberculosis, sleep apnoea, or other lung disease.
